# Amniotic suspension allograft improves pain and function in a rat meniscal tear-induced osteoarthritis model

**DOI:** 10.1186/s13075-022-02750-9

**Published:** 2022-03-04

**Authors:** Kelly A. Kimmerling, Andreas H. Gomoll, Jack Farr, Katie C. Mowry

**Affiliations:** 1 Department of Research & Development, Organogenesis, 2641 Rocky Ridge Lane, Birmingham, AL 35216 USA; 2 Department of Orthopaedic Surgery, Hospital for Special Surgery, New York, NY USA; 3 Knee Preservation and Cartilage Restoration Center, OrthoIndy, Indianapolis, IN USA

**Keywords:** Osteoarthritis, Amniotic suspension allograft, MMT model, Inflammation

## Abstract

**Background:**

Osteoarthritis is a degenerative disease of the knee that affects 250 million people worldwide. Due to the rising incidence of knee replacement and revision surgery, there is a need for a nonsurgical treatment to reduce pain and improve function in patients with knee osteoarthritis. Placental-derived allografts, such as an amniotic suspension allograft (ASA), provide growth factors and cytokines that could potentially modulate the inflammatory environment of osteoarthritis. The purpose of this study was to evaluate the efficacy of ASA in a rat medial meniscal tear (MMT) induced osteoarthritis model through histology, microCT, synovial fluid biomarkers, and behavioral testing.

**Methods:**

Rats underwent MMT surgery at day − 7; at day 0, rats were injected with either ASA, vehicle control, or fibroblast growth factor-18 (FGF18). Behavioral testing, including gait analysis, pain threshold, incapacitance, and knee swelling were evaluated *in-life*, along with histology, microCT analysis of cartilage, and synovial fluid testing post-sacrifice. One MMT cohort was sacrificed at day 10, the other at day 21. A third cohort acted as a safety arm and did not receive MMT surgery; these rats were injected with either vehicle control or ASA and evaluated at day 3 and day 21.

**Results:**

Behavioral testing showed a significant improvement in pain threshold, incapacitance, and gait following an injection of ASA. MicroCT showed significant improvements in cartilage thickness and attenuation at day 10 only, and histology showed no detrimental effects compared to the vehicle control at day 21. Synovial fluid analysis showed a significant increase in anti-inflammatory IL-10. The safety cohort showed no significant differences except for an increase in synovitis at day 21, which could be evidence of a xenogeneic response in this model.

**Conclusions:**

In this study, an injection of ASA was well tolerated with no adverse events. Improvements in pain and function, along with cartilage properties at day 10, were observed. Increases in anti-inflammatory cytokines was also seen, along with no significant cartilage degeneration at day 21 compared to the vehicle control. This study provides evidence for the use of ASA as a nonsurgical treatment for knee OA.

**Supplementary Information:**

The online version contains supplementary material available at 10.1186/s13075-022-02750-9.

## Background

Osteoarthritis (OA) is a degenerative disease that affects the synovial joints, including the knees, hips, and hands [1]. It is estimated that in the USA, 25.6 million adults currently have OA [2], with a higher prevalence in females, older adults, and obese individuals [2, 3]; worldwide, 250 million people are estimated to be affected [1]. Furthermore, the economic burden of OA in the USA is thought to be $200 billion annually, which includes both healthcare costs and lost wages [2]. Of patients with knee OA, adults 50 years or older have a lifetime risk of knee replacement surgery of 10.8% for women and 8.1% for men [4]. In younger patients, the lifetime risk of revision is substantially higher and thought to be between 20 and 35%, with half of these revisions occurring during the first 5 years post-surgery [4]. Due to the cost of treating OA, as well as the increasing volume and risk involved with revision total knee replacement surgery, there is an unmet need for new nonsurgical treatments that would improve pain and function of patients, with the goal of delaying total knee replacement and thus reducing the risk of revision.

One potential nonsurgical approach to improve pain and function in knee OA is the use of placental-derived tissues. Placental tissues include the amnion (inner layer), the chorion (outer layer), umbilical cord, and amniotic fluid [5]. Several combinations of these tissues have been shown to be effective in orthopedic applications both pre-clinically and clinically [5–12]. In the current study, the use of an amniotic suspension allograft (ASA), which contains cells from the amniotic fluid and micronized amniotic membrane, was evaluated in a rat model of OA. Amniotic tissues have been shown to contain several anti-inflammatory cytokines and growth factors, along with factors relevant to OA, including interleukin-1 receptor antagonist (IL-1Ra), tissue inhibitors of matrix metalloproteinases (TIMPs), and interleukin-6 (IL-6) [13].

In this study, we used the rat meniscal tear induced (MMT) model, a well-characterized model for studying the rapid progression of cartilage degeneration and osteophyte formation associated with OA [14, 15]. To determine the efficacy of ASA within this model, we examined pain and function using behavioral assessments *in-life*, cartilage degeneration using histopathology and microCT imaging ex vivo, and cytokine levels in synovial fluid ex vivo. We hypothesized that human-derived ASA would improve pain and function of rats, while modulating the inflammatory environment associated with OA.

## Methods

### Animals and husbandry details

All in-life animal procedures were completed at Bolder BioPATH (Boulder, CO), and protocols were approved by the Bolder BioPATH Institutional Animal Care and Use Committee (IACUC) (Protocol #BBP-008). For the first study (phase I, safety and MMT cohorts), 120 male Lewis rats (Envigo Harlan, Denver, CO) were obtained and acclimated for 7 days prior to the start of the study. Animal weights were between 283 and 340 g at the beginning of the first study. An additional 50 male Lewis rats (Envigo Harlan, Denver, CO) were obtained and acclimated for 7 days prior to phase II of the study. Animal weights were between 292 and 324 g at the beginning of the second study. Rats were housed 2–5 animals per cage on a 12 h/12 h light/dark cycle; Harlan Teklad diet #8640 was fed ad libitum, and unrestricted access to tap water was available during the study.

### Safety study cohorts

**Fig. 1:**
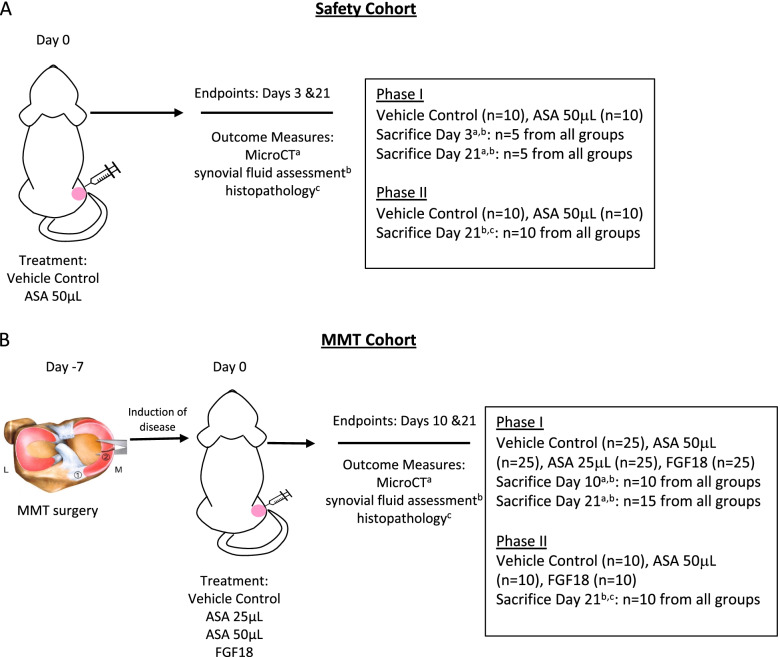
Study design for **A** safety cohort and **B** MMT surgery cohort. ASA, amniotic suspension allograft; MMT, medial meniscal tear; L, lateral; M, medial; 1, cranial meniscotibial ligament; 2, medial meniscus; FGF18, fibroblast growth factor-18; microCT, micro-computed tomography

Twenty rats were injected via intra-articular injection with either 50 μL of the vehicle control (all components of amniotic suspension allograft minus amniotic cells and membrane; negative control) or 50 μL amniotic suspension allograft (ASA (ReNu®, Organogenesis, Birmingham, AL), no dilution) on day 0, as shown in Fig. 1A. Rats were sacrificed on day 3 or day 21; analyses of synovial fluid and joints were conducted to determine safety of the ASA injection.

Due to disarticulation of the joints for microCT, not all histological parameters could be assessed during histopathological grading. As a result of this finding at the end of the first study, an additional twenty male Lewis rats were utilized for the second part of this study; all procedures were identical as described above. In the second study, on day 0, the rats in the safety study arm either received 50 μL vehicle control or 50 μL ASA (no dilution). All animals in the second part of the study were sacrificed 21 days following treatment; synovial fluid and joints were collected for analysis.

### MMT study cohorts

On day − 7, the right knees of 100 rats underwent a medial meniscectomy to induce a medial meniscus tear (MMT) as shown in Fig. 1B. Full details of the surgical procedure have been reported previously [16]. On day 0, rats received a 50 μL injection in the right (operated) knee of one of the following: vehicle control (negative control), 25 μL amniotic suspension allograft (ASA) (1:1 dilution 25 μL ASA (ReNu®; Organogenesis, Birmingham, AL) + 25 μL sterile saline), 50 μL ASA (no dilution), or 5 μg fibroblast growth factor-18 (FGF18) in 50 μL saline [17] (R&D Systems, Minneapolis, MN). Due to the short half-life of FGF18, it was dosed every 7 days; all other injections were given once on day 0.

Behavioral testing was carried out at baseline and during the in-life period following treatment; this schedule can be found in Table 1. Ten days after treatment, 40 rats were sacrificed; the remaining 60 rats were sacrificed 21 days post-treatment. At sacrifice, synovial fluid and knee joints were collected for analysis.

**Table 1:** Behavioral testing schedule for MMT cohort

	Day 0	Day 7	Day 9	Day 14	Day 21
**Behavioral tests**					
Incapacitance testing	X	X			X
Von Frey analysis	X	X			X
Gait analysis			X		
**Other testing**					
Body weight^a^	X	X		X	X
Knee caliper	X	X			X

^a^Body weight was measured for all animals on days − 7 to 0, then weekly afterwards

Due to disarticulation of the joints for microCT, all histological parameters were not able to be assessed during histopathological grading. Because of the increases in synovitis and fibrosis seen in a prior monosodium iodoacetate (MIA) model following injection with ASA [8], complete histological scoring in this study was a key outcome. As a result, an additional thirty male Lewis rats were used; all procedures were identical as described above. On day − 7, 30 rats underwent MMT surgery, and on day 0, rats in the MMT arm received 50 μL injection in the right (operated) knee of one of the following: vehicle control (negative control), 50 μL ASA (no dilution), or 5 μg FGF18 (R&D Systems, Minneapolis, MN). Due to the short half-life of FGF18, it was dosed every 7 days; all other injections were given once on day 0. Rats were all sacrificed 21 days following treatment; synovial fluid and joints were collected for analysis.

### Behavioral testing

Rats underwent behavioral testing (day 0 to day 21), including gait analysis, incapacitance testing, Von Frey analysis, and body weight and caliper measurements of the knee throughout the study (Table 1). Detailed methods for testing are described elsewhere [8]. Briefly, gait testing was done by scoring inked footprint pairs; 0 represents a normal gait, while 6 represents hopping/carrying of the limb. Incapacitance testing measures the weight bearing difference between limbs, with smaller differences indicating less sensitivity to the operated limb. Von Frey analysis measures the pain threshold using filaments ranging from 3.16 to 5.18 grams absolute threshold for rats to the animals’ paw; larger pain threshold scores are representative of less sensitivity with increasing filament size. Digital knee calipers were used to measure swelling of the operated and treated knee. Body weight measurements were taken weekly beginning at day − 7. For all other testing, baseline measurements were taken on day 0 (pre-treatment) and at intervals outlined in Table 1.

### MicroCT analysis of cartilage

At sacrifice, hind limbs were removed, carefully disarticulated (femur/tibia), and fixed with 10% neutral buffered formalin (NBF) for 48 h. Following fixation, limbs were transferred to 70% ethanol and sent to Covance Laboratories Inc. (Greenfield, IN) for micro computed tomography (microCT) analysis of cartilage. Joints were removed from ethanol and stained using 2% phosphotungstic acid (PTA) to enhance the contrast of the articular cartilage. Due to discontinuation of the previously used contrast that is negatively charged and binds to proteoglycans, we used PTA as the contrast agent, which is also negatively charged, has a low pH, and binds to collagen [18]. As a result of using PTA for our study, lower cartilage attenuation correlates with more collagen degeneration, so an increase in cartilage attenuation shows reduced collagen degeneration and reduced cartilage damage. Specimens were placed individually into separate polypropylene tubes for imaging, and then placed back into 70% ethanol upon completion of scanning.

Scanning was done using a SkyScan 1176 (Bruker BioSpin, Billerica, MA), with reconstruction and analysis using the scanner-specific software. X-ray source was 90 kV/25 W, and the detector was 4000 × 2672 pixels. For imaging, the x-ray source was set on 65 kV/25 W with a resolution of 18 μm. Region of interests (ROIs) were divided into either lateral or medial compartments for each sample. Parameters measured included cartilage grayscale, cartilage attenuation, cartilage thickness, and cartilage volume.

### Histopathology analysis of joints

For histopathologic analysis, hind limbs were removed and fixed with 10% neutral buffered formalin (NBF) for 48 h. Following fixation, limbs were transferred to 70% ethanol and sent to HistoTox Labs (Boulder, CO) for histological preparation. Joints were cut in half in the frontal plane and embedded in the same paraffin block for histological assessment. Sections were stained with toluidine blue using standard histological techniques for histopathological grading. The Osteoarthritis Research Society International (OARSI) scoring system was used [19], with minor modifications/additions made to enhance detection of treatment-related changes. Total joint scores and component categories were scored from 0 (normal joint) to 5 (severe degeneration) unless stated otherwise [20].

### Synovial fluid analysis

At sacrifice, synovial fluid was collected via lavage with 100 μL phosphate buffered saline (PBS) containing 1.5% potassium ethylenediaminetetraacetic acid (K_2_EDTA) injected into each knee. Flexion and extension were repeated on each knee, followed by removal of the fluid. Tubes were spun down, and the supernatant was collected and kept at − 80 °C until testing. A Rat Cytokine Array (QAR-CYT-1, RayBiotech, Norcross, GA) and a Rat Inflammation Array (QAR-INF-1, RayBiotech, Norcross, GA) were used to analyze the presence of factors in the synovial fluid. The Rat Cytokine Array panel includes 10 targets (CINC-2, CINC-3, CNTF, Fractalkine, GM-CSF, IL-1α, IL-4, LIX, beta-NGF, and VEGF-A), while the Rat Inflammation Array includes 10 targets (IFN-γ, IL-1α, IL-1β, IL-2, IL-4, IL-6, IL-10, IL-13, MCP-1, and TNF-α); both arrays were run according to manufacturer’s instructions.

### Statistical analysis

All statistics were run using GraphPad Prism 6 (GraphPad Software, La Jolla, CA). For animal behavioral testing, histological data, microCT data, and synovial fluid data, a one-way analysis of variance (ANOVA) was run for each time point, with a Dunnett’s multiple comparisons test to assess statistical significance between groups with the vehicle group as the control. For the safety study cohort, Student’s *t*-test was used to determine significance between the two groups. For synovial fluid assessment, outliers were removed using the interquartile range (IQR) method.

## Results

### Safety cohort

#### Body weight

**Fig. 2:**
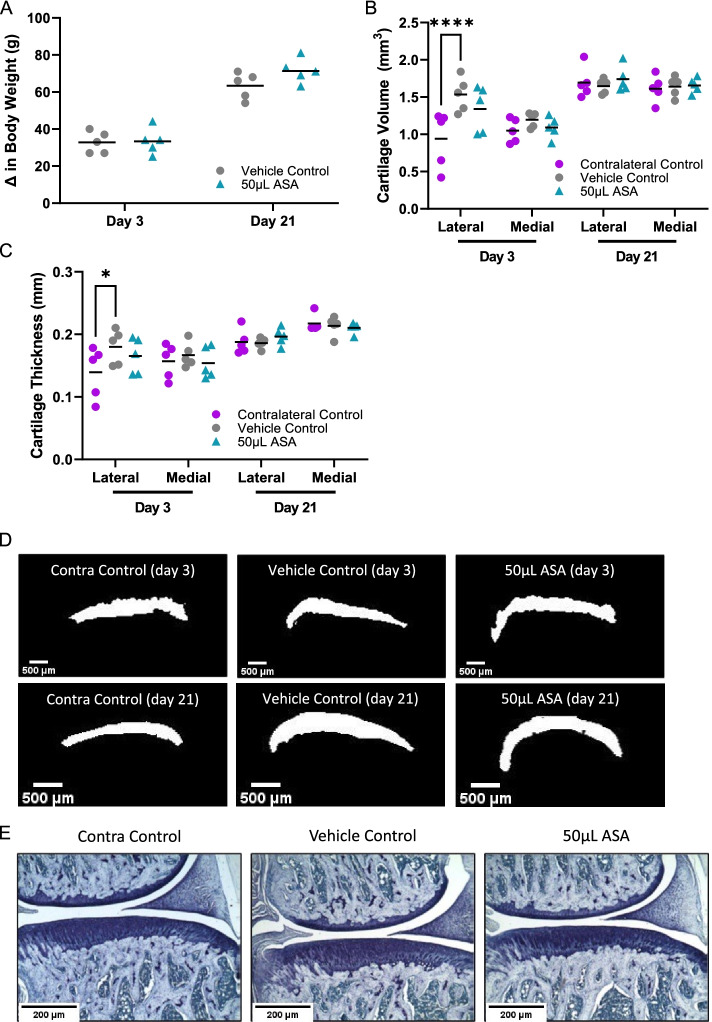
Safety cohort outcomes, including **A** change in body weight at day 3 and day 21, **B** microCT cartilage volume at day 3 and day 21, **C** microCT cartilage thickness at day 3 and day 21, **D** representative microCT images of the medial side for day 3 and day 21, and **E** representative histopathology images of the medial side at day 21 imaged with a × 50 objective. Contra, contralateral; ASA, amniotic suspension allograft

In the safety cohort, we evaluated the effects of an injection of either vehicle control or amniotic suspension allograft (ASA) at both 3 and 21 days. There were no significant changes in body weight between the vehicle control and the 50 μL ASA group at 3 or 21 days (*p* > 0.05; Fig. 2A). Because there was no disease induction, there were no behavioral tests performed as part of this safety cohort.

#### MicroCT analysis of cartilage

Following sacrifice, we examined the cartilage properties using microCT with phosphotungstic acid (PTA) as a contrast agent. There was an incidental finding showing a significant decrease in cartilage volume in the contralateral control compared to the vehicle control on the lateral side at day 3 post-injection (*p* < 0.0001; Fig. 2B); however, there were no other significant differences in the lateral or medial compartment in cartilage volume at day 3 or day 21. Similarly, for cartilage thickness, there was a significant decrease in cartilage thickness in the contralateral control compared to the vehicle control on the lateral side at day 3 post-injection (*p* < 0.05; Fig. 2C); however, there were no other significant differences at either time post-injection compared to the vehicle control. Representative images for each group are shown in Fig. 2D.

#### Histopathology analysis of joints

Histopathology was also completed on the safety cohort; representative images of Toluidine blue staining from each group at 21 days are shown in Fig. 2E. There were no significant differences in the total joint score or the total joint score minus the femur (tibial joint score) between the vehicle control and the 50 μL ASA group (*p* > 0.05; Table 2). Additionally, there was no significant difference between groups for the substantial tibial cartilage degeneration width, bone damage score, bone sclerosis score, osteophyte score, or osteophyte measurement. The synovitis score for the 50 μL ASA group was significantly higher compared to the vehicle control (*p* < 0.01). In ASA-treated joints with synovitis, fragments of ASA were seen around pannus areas, which may be indicative of an inflammatory xenogeneic reaction between the human tissue and rat model. Whole joint views of the images in Fig. 2E can be found in Supplementary Fig. 1A.

**Table 2:** Safety cohort histopathological scoring for each group at day 21

	Vehicle control	50 μL ASA
Total joint score	0.35 ± 0.11	0.40 ± 0.10
Total joint score minus femur	0.35 ± 0.11	0.40 ± 0.10
Cartilage degeneration width (μm)^a^	10.00 ± 10.00	6.00 ± 6.00
Bone damage score	0.00 ± 0.00	0.00 ± 0.00
Bone sclerosis score	0.20 ± 0.13	0.20 ± 0.13
Osteophyte score	0.00 ± 0.00	0.00 ± 0.00
Osteophyte measurement (μm)	0.00 ± 0.00	0.00 ± 0.00
Synovitis	0.20 ± 0.08	1.15 ± 0.21**

Average ± standard error reported; *n* = 10 per group. ** denotes *p* < 0.01 by Mann Whitney test to vehicle control

^a^Defined as substantial tibial cartilage degeneration

#### Synovial fluid analysis

For synovial fluid evaluation at day 3, the 50 μL ASA group had significantly higher levels of IL-1α and beta-nerve growth factor (bNGF) compared to the vehicle control (*p* < 0.05 for both; Table 3). Both levels were not significant by day 21 post-injection. Small but significantly greater levels of interferon gamma (IFN-γ) were seen in the 50 μL ASA group compared to the vehicle control (*p* < 0.001) at day 21 post-injection. Additionally, significantly higher levels of monocyte chemoattractant protein-1 (MCP-1) were found in the 50 μL ASA group compared to the vehicle control (*p* < 0.001).

**Table 3:** Safety cohort biomarkers for each group at days 3 and 21

Target	Concentration (pg/mL)			
	Day 3		Day 21	
	Vehicle control	50 μL ASA	Vehicle control	50 μL ASA
IFN-γ	NT	NT	0.83 ± 0.25	2.58 ± 1.10^
IL-1α	1.19 ± 1.19	41.32 ± 14.88*	74.25 ± 19.37	136.30 ± 34.77
MCP-1	NT	NT	21.76 ± 1.99	46.21 ± 7.91^
bNGF	4.56 ± 1.25	22.29 ± 6.70*	7.72 ± 3.47	4.26 ± 2.71

Average ± standard error reported; *n* = 5–10 per group. *p < 0.05, ^*p* < 0.001 by Dunnett’s multiple comparisons test to vehicle control. Targets not included in the table were not significant compared to vehicle control. *ASA* amniotic suspension allograft, *NT* not tested

### MMT cohort

#### Behavioral testing

**Fig. 3:**
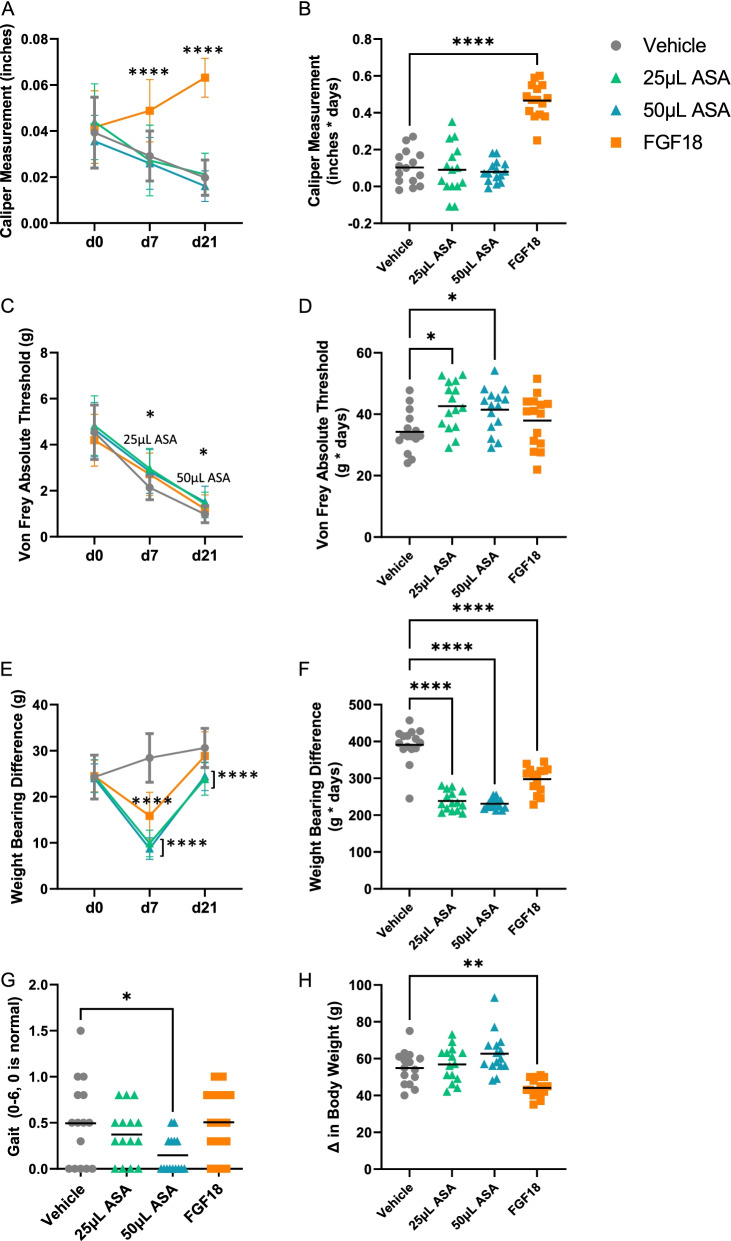
Behavioral testing outcomes for MMT cohort, including **A** knee caliper (swelling) measurement over time, **B** knee caliper measurement area under the curve (AUC), **C** Von Frey absolute threshold (pain) over time, **D** Von Frey AUC, **E** weight bearing difference (incapacitance) over time, **F** incapacitance AUC, **G** gait analysis at day 9, and **H** change in body weight. For all outcomes, average ± standard error reported; *n* = 15 per group. *p < 0.05, **p < 0.01, ****p < 0.0001 by Dunnett’s multiple comparisons test to vehicle control

In the meniscal tear-induced (MMT) OA cohort, we evaluated the effects of an injection of either vehicle control, ASA, or fibroblast growth factor-18 (FGF18) at 10- and 21-days post-treatment. Behavioral testing was done from prior to treatment (baseline, day 0) to sacrifice (day 21). When assessing the effect of injections on swelling, there was a significant increase in knee swelling measured using calipers in the FGF18 group at day 7 (*p* < 0.0001) and day 21 (*p* < 0.0001) post-injection compared to the vehicle control (Fig. 3A). When evaluating swelling measurements as area under the curve (AUC), the only significant increase was seen in the FGF18 group only compared to the vehicle control (*p* < 0.0001; Fig. 3B).

Evaluation of pain threshold using the Von Frey absolute threshold showed that at day 7 post-injection, the 25 μL ASA group had a significantly higher (better) pain threshold compared to the vehicle control (*p* < 0.05; Fig. 3C), and at day 21, the 50 μL ASA group had a significantly higher pain threshold compared to the vehicle control (*p* < 0.05). Looking at the Von Frey AUC to assess the overall response throughout the study, both the 25 μL and 50 μL ASA groups had a significantly higher pain threshold compared to the vehicle control (*p* < 0.05 for both; Fig. 3D).

Weight-bearing difference between the treated and untreated leg, measured by incapacitance, showed that at day 7 post-injection, FGF18, 25 μL ASA, and 50 μL ASA all had significantly lower differences between limbs compared to the vehicle control (*p* < 0.0001 for all; Fig. 3E). At day 21 post-injection, only the 25 μL ASA and 50 μL ASA groups had a significant reduction in weight bearing differences compared to the vehicle control (*p* < 0.0001 for both). When examining the AUC for incapacitance, all treatment groups had significantly lower weight bearing differences compared to the vehicle control (*p* < 0.0001 for all; Fig. 3F).

Gait analysis was completed by evaluating footprints made by inking the back paws. While for all groups the average measurements of gait were relatively low (< 1, normal = 0), this was expected due to the mild nature of the MMT model compared to others [21]. The 50 μL ASA group had a significantly lower gait score compared to the vehicle control at 9 days (*p* < 0.05; Fig. 3G).

When examining average body weight over the course of the study, there were no significant differences between the ASA groups and the vehicle control (*p* > 0.05; Fig. 3H). However, when looking at the change from baseline in body weight at day 21, there was a significant decrease in body weight in the FGF18 group compared to the vehicle control (*p* < 0.01).

#### MicroCT analysis of cartilage

**Fig. 4:**
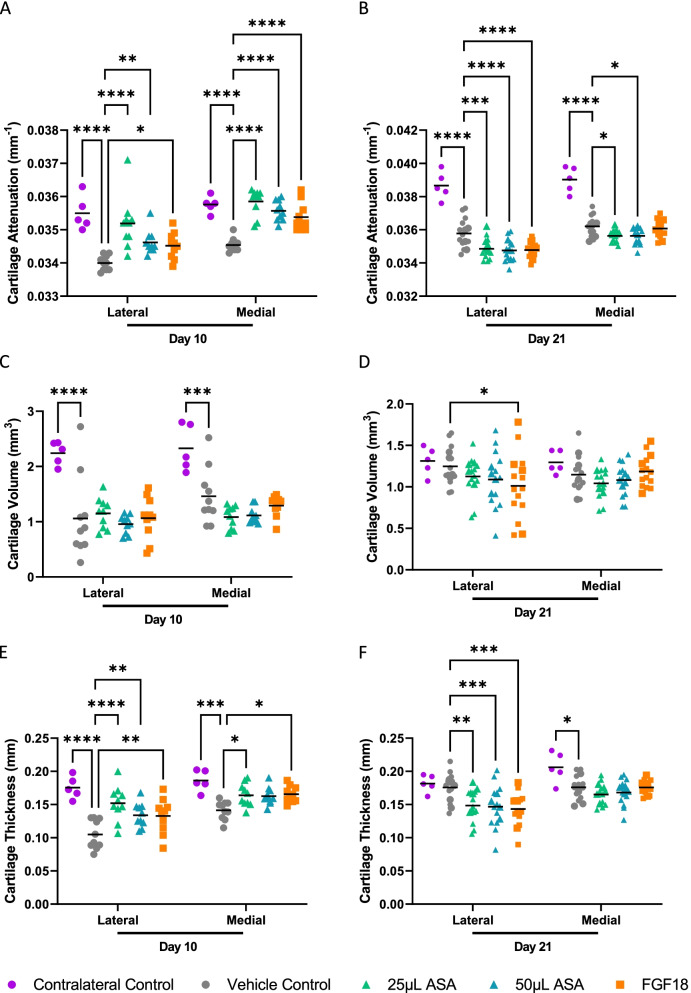
MicroCT outcomes for MMT cohort. Results from **A** cartilage attenuation at day 10, **B** cartilage attenuation at day 21, **C** cartilage volume at day 10, **D** cartilage volume at day 21, **E** cartilage thickness at day 10, and **F** cartilage thickness at day 21. For **A**, **C**, **E**: *n* = 10 per group; for **B**, **D**, **F**: *n* = 15 per group. For all graphs, average ± standard error reported; *p < 0.05, **p < 0.01, ***p < 0.001, and ****p < 0.0001 by Dunnett’s multiple comparisons test to vehicle control. ASA, amniotic suspension allograft; FGF18, fibroblast growth factor-18

After sacrifice at 10 or 21 days, microCT analysis of the cartilage was completed. At day 10, cartilage attenuation on the lateral side showed a significant increase in the 25 μL and 50 μL ASA groups (*p* < 0.0001 for 25 μL ASA, *p* < 0.05 for 50 μL ASA; Fig. 4A), along with FGF18 (*p* < 0.05) and contralateral control (*p* < 0.0001). Medial side cartilage attenuation was significantly increased in all groups compared to the vehicle control (*p* < 0.0001 for all). At 21 days, cartilage attenuation was significantly increased in the contralateral control group (*p* < 0.0001; Fig. 4B) on the lateral side, and significantly decreased in the 25 μL ASA group (*p* < 0.001), 50 μL ASA group (*p* < 0.0001), and FGF18 group (*p* < 0.0001) on the lateral side compared to the vehicle control. Medial side cartilage attenuation was significantly increased in the contralateral control group (*p* < 0.0001) and significantly decreased in the 25 μL and 50 μL ASA groups (*p* < 0.05 for both).

Cartilage volume was significantly increased in the contralateral control compared to the vehicle control on both the lateral and medial sides at day 10 (*p* < 0.0001 and *p* < 0.001, respectively; Fig. 4C). At day 21, there was a significant increase in lateral cartilage volume in the FGF18 group compared to the vehicle control (*p* < 0.05; Fig. 4D). There were no other significant changes in cartilage volume in the ASA or FGF18 groups.

**Fig. 5:**
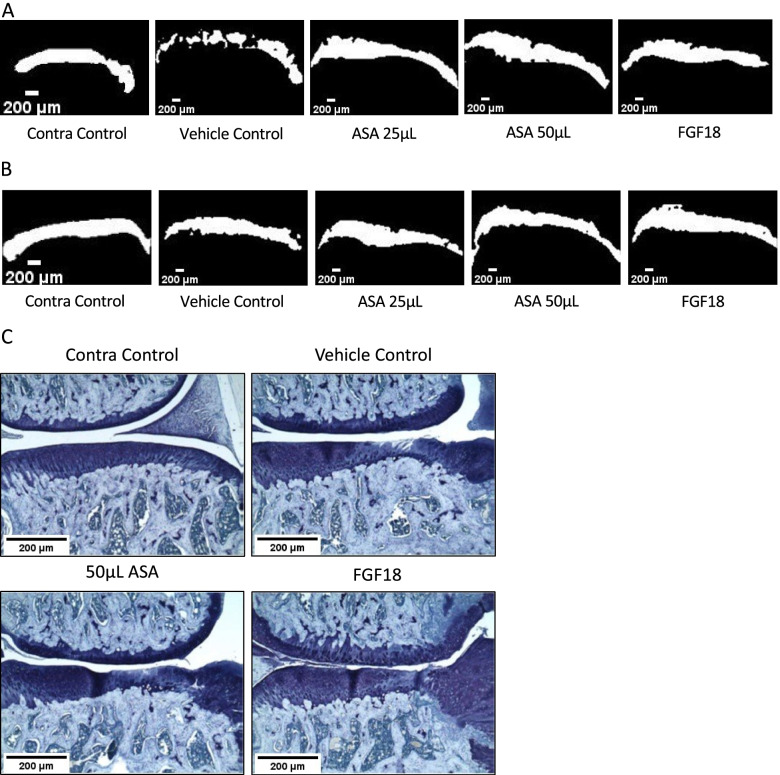
**A** Representative microCT images of the medial side at day 10, **B** representative microCT images of the medial side at day 21, and **C** representative histopathology images for the medial side for each group shown at day 21; × 50 objective. Contra, contralateral; ASA, amniotic suspension allograft; FGF18, fibroblast growth factor-18

Cartilage thickness measurements at day 10 showed a significant increase on the lateral side in the 25 μL ASA (*p* < 0.0001), 50 μL ASA (*p* < 0.001), and the FGF18 (*p* < 0.001) groups compared to the vehicle control group (Fig. 4E), along with the contralateral control (*p* < 0.0001). Medial cartilage thickness was also significantly increased in all treatment groups compared to the vehicle control (25 μL ASA, 50 μL ASA, and FGF18; *p* < 0.01), along with the contralateral control (*p* < 0.0001). At day 21 post-injection, cartilage thickness was significantly decreased on the lateral side in the 25 μL ASA group (*p* < 0.001; Fig. 4F), 50 μL ASA group (*p* < 0.001), and the FGF18 group (*p* < 0.001). On the medial side, cartilage thickness was significantly increased in the contralateral control group only (*p* < 0.05). Representative microCT images at day 10 and day 21 post-injection for each group are shown in Fig. 5A and B, respectively.

#### Histopathology analysis of joints

Histopathology was completed on this MMT cohort at day 21; representative images of joints stained with Toluidine blue are shown in Fig. 5C. Total joint scores in the 50 μL ASA and FGF18 groups were not significant compared to the vehicle control (*p* > 0.05; Table 4); however, when subtracting the femur scores from the total joint score, the FGF18 group was significantly lower than the vehicle control (*p* < 0.05). Substantial tibial cartilage degeneration width was significantly lower in the FGF18 group compared to the vehicle control (*p* < 0.0001), while the bone damage score in the FGF18 group was significantly higher compared to the vehicle control (*p* < 0.001). There were no significant differences in bone sclerosis scores between the three groups. Osteophyte score and osteophyte measurements were significantly increased in the FGF18 group compared to the vehicle control (*p* < 0.001 and *p* < 0.0001, respectively). Finally, synovitis was significantly increased in the FGF18 group (*p* < 0.0001) compared to the vehicle control. Histopathological scoring for each rat is shown in Supplementary Table 1. Whole joint views of the images in Fig. 2E can be found in Supplementary Fig. 1B.

**Table 4:** MMT cohort histopathological scoring for each group at day 21

	Contra control	Vehicle control	50 μL ASA	FGF18
Total joint score	0.20 ± 0.12‡	11.50 ± 0.82	10.07 ± 0.48	9.87 ± 0.26
Total joint score minus femur	0.20 ± 0.12‡	9.93 ± 0.68	8.85 ± 0.33	8.10 ± 0.23*
Cartilage degeneration width (μm)^a^	0.00 ± 0.00‡	670.00 ± 60.18	590.00 ± 36.28	245.00 ± 34.88‡
Bone damage score	0.00 ± 0.00‡	1.73 ± 0.13	1.87 ± 0.28	2.83 ± 0.24^
Bone sclerosis score	0.20 ± 0.20‡	2.40 ± 0.16	2.47 ± 0.09	2.33 ± 0.22
Osteophyte score	0.00 ± 0.00‡	4.00 ± 0.25	3.57 ± 0.34	5.00 ± 0.00^
Osteophyte measurement (μm)	0.00 ± 0.00‡	525.00 ± 24.50	483.33 ± 30.83	988.33 ± 47.27‡
Synovitis	0.00 ± 0.00‡	0.53 ± 0.05	0.92 ± 0.14	3.80 ± 0.13‡

Average ± standard error reported; *n* = 10 per group. *p < 0.05, ^*p* < 0.001, ‡*p* < 0.0001 by Dunnett’s multiple comparisons test to vehicle control. *ASA* amniotic suspension allograft, *FGF18* fibroblast growth factor-18

^a^Defined as substantial tibial cartilage degeneration

#### Synovial fluid analysis

In the MMT cohort, synovial fluid was analyzed for the presence of inflammation-related growth factors and cytokines. Granulocyte-macrophage colony-stimulating factor (GM-CSF) was significantly elevated at day 10 post-injection in the 50 μL ASA group (*p* < 0.05; Table 5); however, by day 21, GM-CSF levels were significantly reduced in the 25 μL ASA and FGF18 groups (*p* < 0.01 and *p* < 0.05, respectively). At day 10, levels of LIX, beta-nerve growth factor (bNGF), and vascular endothelial growth factor-A (VEGF-A) were significantly increased in the FGF18 group compared to the vehicle (*p* < 0.01, *p* < 0.001, and *p* < 0.0001, respectively). At day 21, CINC-2 (*p* < 0.01), fractalkine (*p* < 0.05), IL-4 (*p* < 0.05), IL-6 (*p* < 0.01), LIX (*p* < 0.0001), monocyte chemoattractant protein-1 (MCP-1) (*p* < 0.0001), bNGF (*p* < 0.0001), and VEGF (*p* < 0.0001) levels were significantly elevated in the FGF18 group, while IL-1β levels were significantly reduced (*p* < 0.05). At day 21, levels of IL-2 and IL-10 were significantly elevated in the 50 μL ASA group (*p* < 0.001, *p* < 0.05, and *p* < 0.05, respectively).

**Table 5:** MMT cohort biomarkers for each group at days 10 and 21

Target	Concentration (pg/mL)							
	Day 10				Day 21			
	Vehicle control	25 μL ASA	50 μL ASA	FGF18	Vehicle control	25 μL ASA	50 μL ASA	FGF18
CINC-2	0.00 ± 0.00	0.00 ± 0.00	0.00 ± 0.00	0.00 ± 0.00	0.00 ± 0.00	0.00 ± 0.00	0.00 ± 0.00	1.12 ± 0.48**
Fractalkine	0.00 ± 0.00	0.00 ± 0.00	0.00 ± 0.00	0.00 ± 0.00	0.00 ± 0.00	0.00 ± 0.00	0.00 ± 0.00	76.99 ± 39.24*
GM-CSF	2.18 ± 1.33	2.80 ± 1.64	20.68 ± 6.79*	1.46 ± 0.69	27.50 ± 8.33	4.24 ± 1.51**	17.91 ± 3.86	7.37 ± 1.47*
IL-1β	NT	NT	NT	NT	7.30 ± 3.25	NT	0.35 ± 0.35	0.00 ± 0.00*
IL-2	NT	NT	NT	NT	54.34 ± 18.52	NT	234.60 ± 53.51^	1.84 ± 1.84
IL-4	0.26 ± 0.12	0.25 ± 0.16	0.45 ± 0.18	0.00 ± 0.00	0.17 ± 0.04	0.06 ± 0.02	0.20 ± 0.06	1.64 ± 0.53**
IL-6	NT	NT	NT	NT	3.71 ± 1.45	NT	4.24 ± 2.00	27.72 ± 7.27**
IL-10	NT	NT	NT	NT	20.10 ± 7.99	NT	78.67 ± 23.87*	47.91 ± 17.14
LIX	4.38 ± 1.71	2.08 ± 1.08	8.68 ± 1.73	34.90 ± 12.71**	9.15 ± 2.03	4.37 ± 0.96	5.89 ± 1.16	152.00 ± 27.36‡
MCP-1	NT	NT	NT	NT	79.82 ± 11.36	NT	79.82 ± 12.25	759.90 ± 34.11‡
bNGF	12.85 ± 2.53	17.69 ± 4.46	14.61 ± 3.41	56.44 ± 13.26^	11.09 ± 1.80	16.02 ± 1.38	17.17 ± 1.85	129.70 ± 16.95‡
VEGF	151.40 ± 27.06	224.20 ± 36.66	162.1 ± 17.76	918.80 ± 131.30‡	119.80 ± 10.07	136.20 ± 17.09	173.40 ± 11.36	1234.00 ± 70.32‡

Average ± standard error reported; *n* = 10-15 per group except for IL-4 (*n* = 25). *p < 0.05, **p < 0.01, ^*p* < 0.001, ‡*p* < 0.0001 by Dunnett’s multiple comparisons test to vehicle control. Targets not included in the table were not significant compared to vehicle control. *ASA* amniotic suspension allograft, *FGF18* fibroblast growth factor-18, *NT* not tested

## Discussion

In the safety cohort, there were no significant differences between the ASA and vehicle control group in microCT parameters at 3 days or 21 days. There was an increase in synovitis 21 days post-treatment; in the histopathologic grading, the test article was shown to be present in pannus areas in the synovium. Additionally, there were no observed adverse events in any rats in the current study (safety or MMT).

In the MMT cohort, rats treated with amniotic suspension allograft (ASA) following medial meniscal tear induced (MMT) surgery showed results that were significantly better than vehicle control for pain threshold, weight bearing, and gait. Additionally, 10 days post-OA induction, treatment with ASA resulted in significantly better cartilage thickness and attenuation. Synovial fluid levels of IL-2 and IL-10 were significantly increased at day 21, while GM-CSF levels were significantly increased at day 10 and subsequently decreased by day 21. No significant differences in histopathology scores were found following treatment with ASA.

In a preclinical model of rat MIA, the use of ASA showed significant improvements in pain threshold and weight bearing differences [8], and the results from the present MMT model were consistent with these findings. A clinical pilot safety study demonstrated similar responses in six human patients; a single injection of ASA showed trends towards improved pain and activities of daily living (ADL) subscores of the Knee Injury and Osteoarthritis Outcome Score (KOOS) for up to 1 year [11]. Another single-center clinical study using 1–2 injections of amniotic membrane/umbilical cord (AM/UC) showed a reduction in the Western Ontario and McMaster Universities Osteoarthritis Index (WOMAC) pain and physical function sub-scores up to 24 weeks post-injection [22]. Lastly, a 200-patient randomized controlled clinical trial of a single injection of ASA was recently published [6, 23]; patients receiving ASA had significantly improved pain and patient-reported outcome scores compared to patients receiving hyaluronic acid or saline for up to 12 months post-injection [6, 23].

When looking at cartilage thickness and attenuation from microCT, an injection of ASA showed improvements in cartilage thickness 10 days post-injection after staining with phosphotungstic acid (PTA), along with improvements in cartilage attenuation. Two previous studies, which used micronized amnion and chorion matrix (μ-dHACM) [12] or a particulated AM/UC [9], also showed improvements in cartilage thickness and attenuation compared to controls [9, 12]. Furthermore, early results (day 10) showed significant increases in cartilage thickness and attenuation following treatment with ASA compared to the vehicle control; these changes were not seen at the later time point (day 21). One hypothesis for this observation is that over time, rats experience resolution of pain, leading to increased use of their hind limbs and subsequent cartilage damage. This hypothesis is supported by a previous study by LaBranche et al. who reported that increased weight bearing correlated with increased cartilage damage [24]. In our study, weight bearing difference was significantly lower at day 7; although it remained significantly lower than the vehicle control at day 21, the mean difference had increased, which may explain the cartilage damage observed in the microCT analysis.

Histologically, rats in the safety cohort treated with ASA showed increased synovitis compared to the vehicle control group, with small, non-significant changes in the MMT cohort. These findings are in line with others, where following μ-dHACM injection in the naïve rats and the MMT model, Willett et al. showed the presence of test article in the synovium surrounded by inflammatory infiltrate [12], although they did not perform histopathologic grading of the joints. Raines et al. showed a significant reduction in cartilage degeneration at both 7 and 28 days post-injection with AM/UC compared to saline and a significant reduction of synovitis at 28 days post-injection compared to 7 days post-injection with the lower dose of AM/UC only using the Osteoarthritis Research Society International (OARSI) grading scale [9]. In a chemically induced OA rabbit model, treatment with lyophilized human amniotic membrane showed a significant improvement in Mankin score, which assesses cartilage structure, cellularity, tidemarks, and Safranin-O staining, both 3 and 6 weeks post-injection compared to an injury control [25]. Differences in histological outcomes may be due to the time between MMT surgery and treatment dosing; Raines et al. delayed treatment for 14 days after surgery, Marino-Martinez et al. treated “once the OA model was established,” while the current study delayed treatment by 7 days [9, 25].

Finally, our study utilized biomarkers to determine immune modulating factors within the synovial fluid. In the MMT cohort, IL-2 levels were significantly upregulated 21 days following ASA injection; previously, IL-2 levels have been shown to correlate with advanced OA, as determined by the International Cartilage Repair Society (ICRS) cartilage scale [26]. The lack of improvement in cartilage degeneration at 21 days, along with the increase in IL-2, is evidence for the rapid cartilage degeneration progression in the MMT model. GM-CSF levels were significantly elevated in the 50 μL ASA group 10 days after treatment; these levels were reduced by day 21 and were significantly reduced in the 25 μL ASA group compared to the vehicle control. GM-CSF can act as a pro-inflammatory cytokine and has been linked to IL-1 and TNF-α [27]; due to this finding, anti-GM-CSF treatments have been studied and have shown promising results with a reduction of pain and disease development in a collagenase-induced OA model [28]. Amniotic tissues are known to contain growth factors and cytokines, such as IL-1Ra and soluble TNF receptors, which can modulate the effects of IL-1 and TNF-α, respectively [13, 29]. In this study, levels of anti-inflammatory IL-10 were significantly elevated 21 days post-injection with ASA, similar to results seen from treating MIA-induced rats with ASA [8].

The current study aimed to further characterize the effect of ASA on OA by utilizing the MMT model to examine changes to pain and function as well as cartilage degeneration following ASA treatment. Analysis of cartilage via microCT and histopathology indicated significant improvements in cartilage properties 10 days post-injection, along with the presence of synovitis in the healthy treated joints at 21 days post-ASA injection. Biomarker analysis indicated the presence of some pro-inflammatory cytokines, which was correlated by time to observed histological changes, along with increases in anti-inflammatory cytokines known to modulate the OA environment. Behavioral testing results in the MIA study [8] have been confirmed in this study; additionally, clinical studies with ASA have demonstrated that ASA is a potential clinical treatment for OA with significant improvements seen in pain and function subscores [6].

One limitation of this study was that only male rats were used; previous studies have shown that sex and hormones can affect OA disease severity [30, 31]. To ensure reproducibility between animals in both MMT study phases and therefore limit the total number of animals used overall, only male rats were used within this study. Additionally, the use of human amniotic suspension allograft in a rat model could potentially result in a xenogeneic reaction, as seen in the safety cohort histopathology results. Finally, due to disarticulation of the joints, part of the study had to be repeated in order to be able to conduct full histopathological grading. All efforts were taken to assure uniformity between the original study and additional study cohorts.

Future directions include examining the role of ASA on macrophages in vitro and in vivo, along with further mechanistic exploration into how the immune modulating growth factors and cytokines present in ASA may attenuate the pro-inflammatory environment observed in OA. Furthermore, additional clinical studies evaluating ASA for the treatment of OA are planned.

## Conclusions

Treatment with ASA resulted in improvements in cartilage properties at 10 days post-OA induction; however, these improvements in cartilage were lost by day 21. Additionally, treatment with ASA resulted in increases in anti-inflammatory cytokines in the synovial fluid that potentially suggests modulation of the pro-inflammatory environment associated with OA; pain and function were improved following ASA injection as seen in behavioral testing, which is supported both in a previous animal study and in clinical studies. In sum, these results provide further data supporting the role of ASA as a nonsurgical treatment for knee OA.

## Supplementary Information

**Additional file 1: Supplementary Fig. 1.** Representative histopathology images for the whole joint for each group in the (A) safety cohort and (B) MMT cohort at day 21 imaged with a 4x objective.

**Additional file 2: Supplementary Table 1.** Histopathology scoring for each individual rat at day 21. Contra=contralateral control, ASA=amniotic suspension allograft, MMT=medial meniscal tear, FGF18=fibroblast growth factor-18.

## Data Availability

The datasets analyzed during the current study are available from the corresponding author upon reasonable request.

## Abbreviations

ADL: Activities of daily living
AM/UC: Amniotic membrane/umbilical cord
ANOVA: Analysis of variance
ASA: Amniotic suspension allograft
AUC: Area under the curve
bNGF: Beta-nerve growth factor
FGF18: Fibroblast growth factor-18
GM-CSF: Granulocyte macrophage-colony stimulating factor
IACUC: Institutional Animal Care and Use Committee
IFN-γ: Interferon gamma
IL-1α: Interleukin-1 alpha
IL-1β: Interleukin-1 beta
IL-1Ra: Interleukin-1 receptor antagonist
IL-2: Interleukin-2
IL-4: Interleukin-4
IL-6: Interleukin-6
IL-10: Interleukin-10
K_2_EDTA: Potassium ethylenediaminetetraacetic acid
KOOS: Knee Injury and Osteoarthritis Outcome Score
MCP-1: Monocyte chemoattractant protein-1
MIA: Monosodium iodoacetate
MicroCT: Micro computed tomography
MMT: Medial meniscal tear
NBF: Neutral buffered formalin
OA: Osteoarthritis
OARSI: Osteoarthritis Research Society International
PGE_2_: Prostaglandin E_2_
PTA: Phosphotungstic acid
ROI: Region of interest
TIMPs: Tissue inhibitor of matrix metalloproteinases
TNF-α: Tumor necrosis factor alpha
μ-dHACM: Micronized amnion and chorion matrix
VEGF: Vascular endothelial growth factor
WOMAC: Western Ontario and McMasters University Osteoarthritis Index

## References

[1] O’Neill TW, McCabe PS, McBeth J (2018). Update on the epidemiology, risk factors and disease outcomes of osteoarthritis. Best Pract Res Clin Rheumatol.

[2] Zhao X, Shah D, Gandhi K, Wei W, Dwibedi N, Webster L (2019). Clinical, humanistic, and economic burden of osteoarthritis among noninstitutionalized adults in the United States. Osteoarthr Cartil.

[3] Van Manen MD, Nace J, Mont MA (2012). Management of primary knee osteoarthritis and indications for total knee arthroplasty for general practitioners. J Am Osteopath Assoc.

[4] Price AJ, Alvand A, Troelsen A, Katz JN, Hooper G, Gray A (2018). Knee replacement. Lancet..

[5] McIntyre JA, Jones IA, Danilkovich A, Vangsness CT (2018). The placenta: applications in orthopaedic sports medicine. Am J Sports Med.

[6] Farr J, Gomoll AH, Yanke AB, Strauss EJ, Mowry KC. A randomized controlled single-blind study demonstrating superiority of amniotic suspension allograft injection over hyaluronic acid and saline control for modification of knee osteoarthritis symptoms. J Knee Surg. 2019;32(11):1143–54. Available from: http://www.thieme-connect.de/DOI/DOI?10.1055/s-0039-1696672. 10.1055/s-0039-1696672 31533151

[7] Heckmann N, Auran R, Mirzayan R (2016). Application of amniotic tissue in orthopedic surgery. Am J Orthop (Belle Mead NJ).

[8] Kimmerling KA, Gomoll AH, Farr J, Mowry KC (2020). Amniotic suspension allograft modulates inflammation in a rat pain model of osteoarthritis. J Orthop Res.

[9] Raines AL, Shih MS, Chua L, Su CW, Tseng SCG, O’Connell J (2017). Efficacy of particulate amniotic membrane and umbilical cord tissues in attenuating cartilage destruction in an osteoarthritis model. Tissue Eng Part A.

[10] Riboh JC, Saltzman BM, Yanke AB, Cole BJ (2016). Human amniotic membrane-derived products in sports medicine: basic science, early results, and potential clinical applications. Am J Sports Med.

[11] Vines JB, Aliprantis AO, Gomoll AH, Farr J (2016). Cryopreserved amniotic suspension for the treatment of knee osteoarthritis. J Knee Surg.

[12] Willett NJ, Thote T, Lin ASP, Moran S, Raji Y, Sridaran S (2014). Intra-articular injection of micronized dehydrated human amnion/chorion membrane attenuates osteoarthritis development. Arthritis Res Ther.

[13] McQuilling JP, Vines JB, Kimmerling KA, Mowry KC (2017). Proteomic comparison of amnion and chorion and evaluation of the effects of processing on placental membranes. Wounds a Compend Clin Res Pract.

[14] Bendele AM (2001). Animal models of osteoarthritis. J Musculoskelet Neuronal Interact.

[15] Bendele AM, Bronner F, Farach-Carson MC (2007). Animal models. Bone and osteoarthritis.

[16] Janusz MJ, Bendele AM, Brown KK, Taiwo YO, Hsieh L, Heitmeyer SA (2002). Induction of osteoarthritis in the rat by surgical tear of the meniscus: inhibition of joint damage by a matrix metalloproteinase inhibitor. Osteoarthr Cartil.

[17] Moore EE, Bendele AM, Thompson DL, Littau A, Waggie KS, Reardon B (2005). Fibroblast growth factor-18 stimulates chondrogenesis and cartilage repair in a rat model of injury-induced osteoarthritis. Osteoarthr Cartil.

[18] Karhula SS, Finnilä MA, Lammi MJ, Ylärinne JH, Kauppinen S, Rieppo L (2017). Effects of articular cartilage constituents on phosphotungstic acid enhanced micro-computed tomography. PLoS One.

[19] Gerwin N, Bendele AM, Glasson S, Carlson CS (2010). The OARSI histopathology initiative - recommendations for histological assessments of osteoarthritis in the rat. Osteoarthr Cartil.

[20] TenBroek EM, Yunker L, Nies MF, Bendele AM. Randomized controlled studies on the efficacy of antiarthritic agents in inhibiting cartilage degeneration and pain associated with progression of osteoarthritis in the rat. Arthritis Res Ther. 2016;18:24. 10.1186/s13075-016-0921-5 PMC4721142 26794830

[21] Aüllo-Rasser G, Dousset E, Roffino S, Zahouani H, Lecurieux-Clerville R, Argenson JN (2020). Early-stage knee OA induced by MIA and MMT compared in the murine model via histological and topographical approaches. Sci Rep.

[22] Castellanos R, Tighe S (2019). Injectable amniotic membrane/umbilical cord particulate for knee osteoarthritis: a prospective, single-center pilot study. Pain Med.

[23] Gomoll AH, Farr J, Cole BJ, Flanigan DC, Lattermann C, Mandelbaum BR (2021). Safety and efficacy of an amniotic suspension allograft injection over 12 months in a single-blinded, randomized controlled trial for symptomatic osteoarthritis of the knee. Arthrosc - J Arthrosc Relat Surg.

[24] LaBranche TP, Bendele AM, Omura BC, Gropp KE, Hurst SI, Bagi CM (2017). Nerve growth factor inhibition with tanezumab influences weight-bearing and subsequent cartilage damage in the rat medial meniscal tear model. Ann Rheum Dis.

[25] Marino-Martínez IA, Martínez-Castro AG, Peña-Martínez VM, Acosta-Olivo CA, Vílchez-Cavazos F, Guzmán-López A (2019). Human amniotic membrane intra-articular injection prevents cartilage damage in an osteoarthritis model. Exp Ther Med.

[26] Thomas Vangsness J, Burke WS, Narvy SJ, MacPhee RD, Fedenko AN (2011). Human knee synovial fluid cytokines correlated with grade of knee osteoarthritis: a pilot study. Bull NYU Hosp Jt Dis.

[27] Hamilton JA (2008). Colony-stimulating factors in inflammation and autoimmunity. Nat Rev Immunol.

[28] Lee KMC, Prasad V, Achuthan A, Fleetwood AJ, Hamilton JA, Cook AD (2020). Targeting GM-CSF for collagenase-induced osteoarthritis pain and disease in mice. Osteoarthr Cartil.

[29] Kupferminc MJ, Peaceman AM, Aderka D, Wallach D, Peyser MR, Lessing JB (1995). Soluble tumor necrosis factor receptors in maternal plasma and second-trimester amniotic fluid. Am J Obstet Gynecol.

[30] Macrini TE, Coan HB, Levine SM, Lerma T, Saks CD, Araujo DJ (2013). Reproductive status and sex show strong effects on knee OA in a baboon model. Osteoarthr Cartil.

[31] Ma HL, Blanchet TJ, Peluso D, Hopkins B, Morris EA, Glasson SS (2007). Osteoarthritis severity is sex dependent in a surgical mouse model. Osteoarthr Cartil.

