# Supplementary figures and images for: Amniotic suspension allograft improves pain and function in a rat meniscal tear-induced osteoarthritis model
Source: Arthritis Res Ther. 2022 Mar 4;24:63. doi: 10.1186/s13075-022-02750-9 (PMC8895852; doi:10.1186/s13075-022-02750-9)

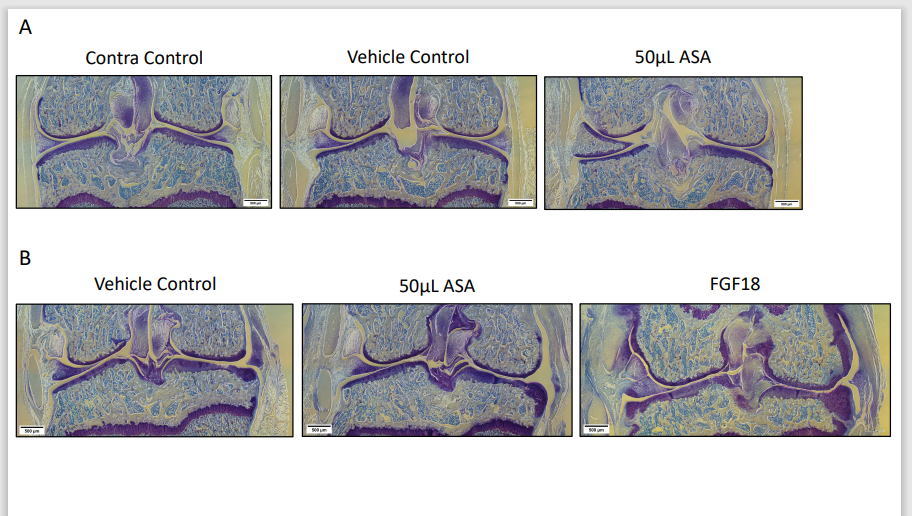

Supplement: Supplementary file 1 — Additional file 1: Supplementary Fig. 1. Representative histopathology images for the whole joint for each group in the (A) safety cohort and (B) MMT cohort at day 21 imaged with a 4x objective. [file 13075_2022_2750_MOESM1_ESM.docx]
