# Supplementary material for: Amniotic suspension allograft improves pain and function in a rat meniscal tear-induced osteoarthritis model
Source: Arthritis Res Ther. 2022 Mar 4;24:63. doi: 10.1186/s13075-022-02750-9 (PMC8895852; doi:10.1186/s13075-022-02750-9)
Supplement: Supplementary file 2 — Additional file 2: Supplementary Table 1. Histopathology scoring for each individual rat at day 21. Contra=contralateral control, ASA=amniotic suspension allograft, MMT=medial meniscal tear, FGF18=fibroblast growth factor-18. [file 13075_2022_2750_MOESM2_ESM.docx]

**Supplementary Table 1:** Histopathology scoring for each individual rat at day 21. Contra=contralateral control, ASA=amniotic suspension allograft, MMT=medial meniscal tear, FGF18=fibroblast growth factor-18

| Rat #/Group | Total Joint Score Minus Femur | Total Joint Score | Cartilage Degeneration Width (μm)^1^ | Bone Damage Score | Bone Sclerosis Score | Osteophyte Score | Osteophyte Measurement (µm) | Synovitis |
| --- | --- | --- | --- | --- | --- | --- | --- | --- |
| **Safety Cohort (no MMT)** | | | | | | | | |
| 1/Contra Control | 0.00 | 0.00 | 0 | 0.0 | 0.0 | 0.0 | 0 | 0.0 |
| 2/Contra Control | 0.00 | 0.00 | 0 | 0.0 | 0.0 | 0.0 | 0 | 0.0 |
| 3/Contra Control | 0.00 | 0.00 | 0 | 0.0 | 0.0 | 0.0 | 0 | 0.0 |
| 4/Contra Control | 1.00 | 1.00 | 0 | 0.0 | 1.0 | 0.0 | 0 | 0.0 |
| 5/Contra Control | 1.00 | 1.00 | 0 | 0.0 | 0.0 | 0.0 | 0 | 0.0 |
| 1/Vehicle Control | 0.50 | 0.50 | 0 | 0.0 | 0.0 | 0.0 | 0 | 0.5 |
| 2/Vehicle Control | 0.00 | 0.00 | 0 | 0.0 | 0.0 | 0.0 | 0 | 0.5 |
| 3/Vehicle Control | 0.00 | 0.00 | 0 | 0.0 | 0.0 | 0.0 | 0 | 0.5 |
| 4/Vehicle Control | 0.50 | 0.50 | 0 | 0.0 | 0.0 | 0.0 | 0 | 0.5 |
| 5/Vehicle Control | 0.50 | 0.50 | 0 | 0.0 | 0.0 | 0.0 | 0 | 0.0 |
| 6/Vehicle Control | 0.00 | 0.00 | 0 | 0.0 | 1.0 | 0.0 | 0 | 0.0 |
| 7/Vehicle Control | 0.50 | 0.50 | 0 | 0.0 | 0.0 | 0.0 | 0 | 0.0 |
| 8/Vehicle Control | 0.00 | 0.00 | 0 | 0.0 | 0.0 | 0.0 | 0 | 0.0 |
| 9/Vehicle Control | 1.00 | 1.00 | 100 | 0.0 | 1.0 | 0.0 | 0 | 0.0 |
| 10/Vehicle Control | 0.50 | 0.50 | 0 | 0.0 | 0.0 | 0.0 | 0 | 0.0 |
| 1/50μL ASA | 0.50 | 0.50 | 0 | 0.0 | 0.0 | 0.0 | 0 | 1.0 |
| 2/50μL ASA | 0.50 | 0.50 | 0 | 0.0 | 0.0 | 0.0 | 0 | 0.0 |
| 3/50μL ASA | 1.00 | 1.00 | 60 | 0.0 | 0.0 | 0.0 | 0 | 2.0 |
| 4/50μL ASA | 0.50 | 0.50 | 0 | 0.0 | 0.0 | 0.0 | 0 | 1.0 |
| 5/50μL ASA | 0.50 | 0.50 | 0 | 0.0 | 0.0 | 0.0 | 0 | 1.0 |
| 6/50μL ASA | 0.00 | 0.00 | 0 | 0.0 | 0.0 | 0.0 | 0 | 1.0 |
| 7/50μL ASA | 0.50 | 0.50 | 0 | 0.0 | 1.0 | 0.0 | 0 | 1.0 |
| 8/50μL ASA | 0.00 | 0.00 | 0 | 0.0 | 1.0 | 0.0 | 0 | 2.0 |
| 9/50μL ASA | 0.50 | 0.50 | 0 | 0.0 | 0.0 | 0.0 | 0 | 0.5 |
| 10/50μL ASA | 0.00 | 0.00 | 0 | 0.0 | 0.0 | 0.0 | 0 | 2.0 |
| **MMT Cohort** | | | | | | | | |
| 1/Vehicle Control | 6.83 | 8.17 | 400 | 2.0 | 2.0 | 3.0 | 400 | 0.5 |
| 2/Vehicle Control | 8.83 | 9.50 | 517 | 2.0 | 3.3 | 4.3 | 550 | 0.5 |
| 3/Vehicle Control | 10.33 | 13.00 | 667 | 1.3 | 2.3 | 4.7 | 600 | 0.5 |
| 4/Vehicle Control | 10.67 | 12.33 | 767 | 2.7 | 2.7 | 4.0 | 550 | 0.5 |
| 5/Vehicle Control | 11.83 | 13.50 | 800 | 1.3 | 2.0 | 5.0 | 617 | 0.5 |
| 6/Vehicle Control | 12.17 | 13.33 | 867 | 1.7 | 2.0 | 4.0 | 533 | 1.0 |
| 7/Vehicle Control | 9.33 | 10.67 | 750 | 1.3 | 2.0 | 3.0 | 417 | 0.5 |
| 8/Vehicle Control | 9.00 | 10.67 | 500 | 1.7 | 2.7 | 4.0 | 533 | 0.5 |
| 9/Vehicle Control | 13.33 | 16.00 | 967 | 1.7 | 3.0 | 5.0 | 600 | 0.3 |
| 10/Vehicle Control | 7.00 | 7.83 | 467 | 1.7 | 2.0 | 3.0 | 450 | 0.5 |
| 1/50μL ASA | 10.17 | 10.67 | 567 | 4.0 | 2.0 | 5.0 | 633 | 1.0 |
| 2/50μL ASA | 9.67 | 11.33 | 533 | 1.3 | 2.3 | 4.0 | 500 | 0.8 |
| 3/50μL ASA | 8.83 | 9.67 | 517 | 1.7 | 2.3 | 4.0 | 517 | 0.5 |
| 4/50μL ASA | 9.33 | 10.33 | 617 | 1.7 | 2.3 | 4.0 | 517 | 1.0 |
| 5/50μL ASA | 7.67 | 7.83 | 600 | 2.0 | 2.7 | 3.0 | 433 | 0.8 |
| 6/50μL ASA | 9.67 | 10.67 | 533 | 1.3 | 2.7 | 5.0 | 617 | 0.5 |
| 7/50μL ASA | 9.83 | 12.17 | 767 | 1.7 | 2.3 | 3.7 | 500 | 1.0 |
| 8/50μL ASA | 7.17 | 7.50 | 367 | 1.0 | 2.3 | 3.0 | 417 | 0.5 |
| 9/50μL ASA | 8.17 | 9.17 | 700 | 1.3 | 2.7 | 2.0 | 350 | 2.0 |
| 10/50μL ASA | 8.00 | 11.33 | 700 | 2.7 | 3.0 | 2.0 | 350 | 1.0 |
| 1/FGF18 | 8.00 | 10.67 | 217 | 1.7 | 1.0 | 5.0 | 1133 | 4.0 |
| 2/FGF18 | 7.00 | 8.17 | 100 | 1.7 | 1.3 | 5.0 | 1100 | 4.0 |
| 3/FGF18 | 9.17 | 11.00 | 400 | 4.0 | 2.7 | 5.0 | 883 | 4.0 |
| 4/FGF18 | 8.17 | 9.67 | 250 | 2.7 | 2.0 | 5.0 | 900 | 4.0 |
| 5/FGF18 | 8.83 | 9.67 | 350 | 3.0 | 2.3 | 5.0 | 900 | 4.0 |
| 6/FGF18 | 8.50 | 10.67 | 317 | 3.7 | 2.7 | 5.0 | 967 | 4.0 |
| 7/FGF18 | 8.67 | 9.67 | 333 | 3.3 | 3.0 | 5.0 | 1000 | 3.0 |
| 8/FGF18 | 7.83 | 10.00 | 217 | 2.3 | 2.7 | 5.0 | 800 | 4.0 |
| 9/FGF18 | 7.83 | 9.33 | 217 | 3.0 | 2.7 | 5.0 | 900 | 4.0 |
| 10/FGF18 | 7.00 | 9.83 | 50 | 3.0 | 3.0 | 5.0 | 1300 | 3.0 |

^1^Defined as substantial tibial cartilage degeneration.
